# Food Authentication: Techniques, Trends and Emerging Approaches (Second Issue)

**DOI:** 10.3390/foods11131926

**Published:** 2022-06-28

**Authors:** Raúl González-Domínguez

**Affiliations:** 1 Agrifood Laboratory, Faculty of Experimental Sciences, University of Huelva, 21007 Huelva, Spain; raul.gonzalez@dqcm.uhu.es; Tel.: +34-959-219-975; 2 International Campus of Excellence CeiA3, University of Huelva, 21007 Huelva, Spain

The authentication of foods and beverages is a very current topic of great interest for all the actors involved in the food chain, including the food industry, consumers, and food science researchers. Food authenticity covers many different aspects related to mislabeling, adulteration, and misleading claims about origin, production method, or processing technologies. As many factors may affect the chemical composition of foods (e.g., geographical origin, variety or breed, conditions of cultivation, breeding and/or feeding), the implementation of accurate, robust, and high-throughput analytical methods is needed to assess their authenticity and traceability and, consequently, to guarantee their safety and quality in terms of organoleptic, nutritional, and bioactive characteristics. For these purposes, multiple analytical tools can be employed in combination with advanced chemometrics, such as spectroscopic and chromatographic techniques, DNA-based methods, and state-of-the-art omics approaches. In this context, the journal *Foods* launched the Special Issue “Food Authentication: Techniques, Trends and Emerging Approaches” in 2020 to gather research papers and review articles dealing with the development and application of analytical techniques and emerging approaches in food authentication [1]. Considering the success and popularity of this Special Issue, we now release a Second Issue comprising 10 valuable scientific contributions, including 1 review article, 1 commentary article, and 8 original research articles.

Fanelli et al. reviewed the most widely used DNA-based molecular techniques for authenticating and tracing fresh and processed agri-food products, from traditional molecular marker-based methods (e.g., single nucleotide polymorphisms) to more recent single region approaches (e.g., DNA barcoding, isothermal amplification-based methods) and next-generation-sequencing-based methods (e.g., DNA metabarcoding) [2]. Herein, an overview of recent advances and applications and an exhaustive comparison of the main advantages and limitations of each molecular method are provided. The importance of properly controlling the mislabeling and adulteration of digested coffees is reported in another commentary article [3]. The authors state that a great part of the coffee labelled as “Kopi Luwak” that can be found in the market is frequently adulterated with undigested coffee beans. Furthermore, they propose that the chemical and organoleptic characteristics of this specialty coffee could be majorly allocated to the diet of the civet cats (i.e., Coffea species, ripeness) rather than to changes caused by digestion.

Many of the original research articles published in this Special Issue revolve around the implementation of low-cost, ecofriendly, and non-destructive spectroscopic methods as a reliable alternative to traditional chemical-based analytical approaches for simple and rapid food authentication. In this respect, González-Domínguez et al. described the potential of ultraviolet-visible spectroscopy in combination with multivariate statistical tools to discriminate Spanish wine vinegars produced under three Protected Designations of Origin (PDO), namely, “Jerez”, “Condado de Huelva”, and “Montilla-Moriles” [4]. Additionally, regression analysis demonstrated that spectral data could accurately predict the physicochemical and functional properties of vinegars, particularly their total phenolic content and antioxidant activity. Similarly, Fourier-transform infrared (FT-IR) spectroscopy was also found to be a practical methodology for the classification of Sherry vinegars according to their origin [5]. Statistical modelling was applied to develop a characteristic spectral fingerprint (“spectralprint”) by selecting the most important variables, which enabled the rapid, reliable, and uncomplicated differentiation of vinegar samples depending on the starting wine. The same spectroscopic approach was employed in another study to discriminate Asian red pepper samples based on their geographical origin [6]. The four most significant peak variables from second-derivative FT-IR spectral data were selected as discriminant indicator variables, and their origin-specific ranges were set. These indicator ranges were able of successfully classifying all the samples under investigation. The last paper published in this Special Issue focused on the application of spectroscopic methods describes the utility of vibrational spectroscopy to predict the fatty acid profile of potato chips with the aim of authenticating the type of oil used in manufacturing [7]. Fatty acids were analyzed by gas chromatography with flame ionization detection (GC-FID), and spectral data were collected using Raman and near-infrared (NIR) sensors. Interestingly, pattern recognition analysis enabled the prediction of the major fatty acid composition and the detection of mislabeling issues. 

As an alternative approach, other authors reported the use of chromatography-based techniques for authenticity and traceability purposes. León-Camacho and Pérez-Camino developed a new supported liquid extraction (SLE) method that, in combination with high-performance liquid chromatography (HPLC) and GC-FID, simplifies the isolation and quantification of the unsaponifiable fraction from fats and oils [8]. This procedure is easier, less time-consuming, and reduces the volume of solvents and reagents compared to traditional liquid–liquid extraction. Furthermore, this method ensured the efficient removal of fatty acids, thereby avoiding possible interferences during GC quantification and facilitating the determination of sterols and triterpenic dialcohols. In another study, untargeted fingerprinting analysis based on high-performance liquid chromatography with ultraviolet and fluorescence detection (HPLC-UV-FLD) was employed to detect common adulterants in coffee, namely, chicory, barley, and flours [9]. In combination with advanced chemometric tools, the methodology provided appropriate performance to detect and quantify adulterant levels down to 15% with good calibration and prediction errors.

The determination of alkaline phosphatase was also proposed as a potential marker for controlling cheeses produced under PDOs [10]. Alkaline phosphatase values in Pecorino Siciliano PDO samples were found to be strongly affected by the type of milk used during cheese production (i.e., raw milk vs. pasteurized milk) and by the temperature during cooking. This variability, probably because of the high craftsmanship, did not permit the researchers to establish clear ranges for discriminating cheeses depending on the production process. Alternatively, Quek et al. compared the overall performance of five DNA extraction procedures for identifying the origin of an edible bird’s nest [11]. They concluded that a hybrid method, combining conventional SDS and a commercial kit (SDS/Qiagen), was the most suitable in terms of speed and cost-effectiveness.

In conclusion, the Special Issue “Food Authentication: Techniques, Trends and Emerging Approaches (Second Issue)” highlights the crucial importance of combining state-of-the-art analytical techniques with advanced statistical approaches with the aim of obtaining deeper insights into food composition and the discovery of novel authenticity indicators.
